# Medical graphics to improve patient understanding and anxiety in elderly and cognitively impaired patients scheduled for transcatheter aortic valve implantation (TAVI)

**DOI:** 10.1007/s00392-023-02352-8

**Published:** 2023-12-20

**Authors:** A. Brand, C. Hornig, C. Crayen, A. Hamann, S. Martineck, D. M. Leistner, H. Dreger, S. Sündermann, A. Unbehaun, M. Sherif, A. Haghikia, S. Bischoff, J. Lueg, Y. Kühnle, O. Paul, S. Squier, K. Stangl, V. Falk, U. Landmesser, V. Stangl

**Affiliations:** 1 Department of Cardiology, Angiology and Intensive Care Medicine, Deutsches Herzzentrum der Charité, Campus Charité Mitte, Berlin, Germany; 2 Department of Cardiology, Angiology and Intensive Care Medicine, Deutsches Herzzentrum der Charité, Campus Benjamin Franklin, Berlin, Germany; 3 DZHK (German Centre for Cardiovascular Research), partner Site Berlin, Berlin, Germany; 4 Department of Education and Psychology, Freie Universität Berlin, Habelschwerdter Allee 45, 14195 Berlin, Germany; 5 Mintwissen-Science Communication Agency and Publishing House, Paulusstr. 11, 40237 Düsseldorf, Germany; 6 Kollwitzstraße 52, 10405 Berlin, Germany; 7 Department of Cardiothoracic and Vascular Surgery, Deutsches Herzzentrum der Charité, Berlin, Germany; 8 Department of Cardiology, Angiology and Intensive Care Medicine, Deutsches Herzzentrum der Charité, Campus Virchow Klinikum, Berlin, Germany; 9 Brill Professor Emeritus of English and Women’s, Gender and Sexuality Studies, The Pennsylvania State University, University Park, PA 16802 USA; 10 Department of Cardiology, Angiology and Intensive Care Medicine, Goethe University Hospital, Universitäres Herz- und Gefässzentrum Frankfurt, Frankfurt am Main, 60590 Frankfurt, Germany; 11 DZHK (German Centre for Cardiovascular Research), partner site Rhein-Main, Munich, Germany

**Keywords:** Informed consent, TAVI, TAVR, Cognitive dysfunction, Medical graphics, Comic

## Abstract

**Background:**

Anxiety and limited patient comprehension may pose significant barriers when informing elderly patients about complex procedures such as transcatheter aortic valve implantation (TAVI).

**Objectives:**

We aimed to evaluate the utility of medical graphics to improve the patient informed consent (IC) before TAVI.

**Methods:**

In this prospective, randomized dual center study, 301 patients were assigned to a patient brochure containing medical graphics (Comic group, *n* = 153) or sham information (Control group, *n* = 148) on top of usual IC. Primary outcomes were patient understanding of central IC-related aspects and periprocedural anxiety assessed by the validated Spielberger State Trait Anxiety Inventory (STAI), both analyzed by cognitive status according to the Montreal Cognitive Assessment (MoCA).

**Results:**

Patient understanding was significantly higher in the Comic group [mean number of correct answers 12.8 (SD 1.2) vs. 11.3 (1.8); mean difference 1.5 (95% CI 1.2–1.8); *p* < 0.001]. This effect was more pronounced in the presence of cognitive dysfunction (MoCA < 26) [12.6 (1.2) in the Comic vs. 10.9 (1.6) in the Control group; mean difference 1.8 (1.4–2.2), *p* < 0.001]. Mean STAI score declined by 5.7 (95% CI 5.1–6.3; *p* < 0.001) in the Comic and 0.8 points (0.2–1.4; *p* = 0.015) in the Control group. Finally, mean STAI score decreased in the Comic group by 4.7 (3.8–5.6) in cognitively impaired patients and by 6.6 (95% CI 5.8 to 7.5) in patients with normal cognitive function (*p* < 0.001 each).

**Conclusions:**

Our results prove beneficial effects for using medical graphics to inform elderly patients about TAVI by improving patient understanding and reducing periprocedural anxiety (DRKS00021661; 23/Oct/2020).

**Graphical Abstract:**

Medical graphics entailed significant beneficial effects on the primary endpoints, patient understanding and periprocedural anxiety, compared to the usual patient informed consent (IC) procedure. Patient understanding of IC-related aspects was significantly higher in the Comic group, with a more pronounced benefit in patients with cognitive impairment (*p* for IC method and cognitive status < 0.001, respectively; *p* for IC method x MoCA category interaction = 0.017). There further was a significant decline of periprocedural anxiety in patients with and without cognitive impairment (*p* for IC method x measuring time point < 0.001; *p* for IC method x MoCA category x measuring time point interaction = 0.018)

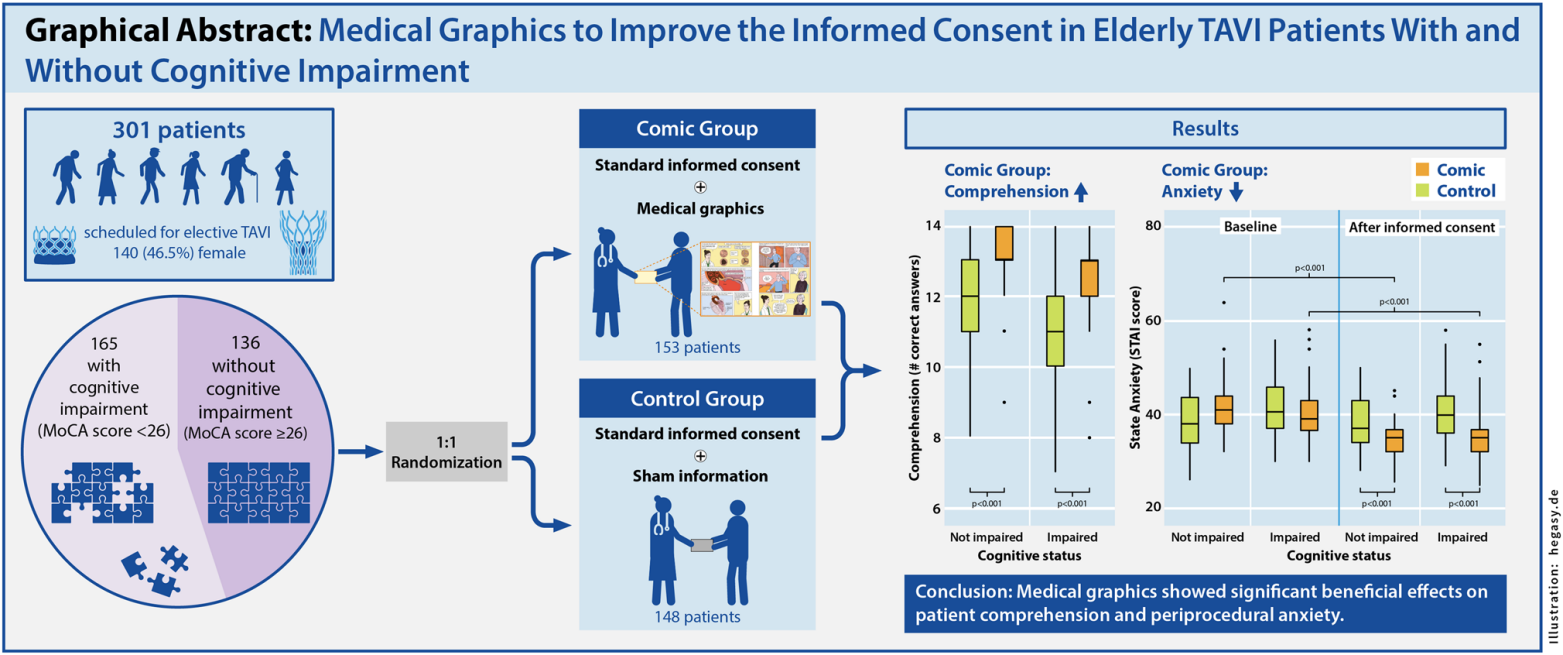

## Introduction

With an estimated pooled prevalence of 3.4% in the general elderly population [1], aortic valve stenosis (AS) accounts for the most common heart valve disease in Europe and North America [2]. Transcatheter aortic valve implantation (TAVI) has become the treatment of choice in elderly patients [3]. Even though current ESC/EACTS Guidelines on valvular heart disease stress the importance of a “patient-centered approach” and advise to consider the “wishes of a well-informed patient” [3], little scientific effort has been undertaken so far to evaluate potential benefits of alternative informed consent (IC) approaches before TAVI. As a consequence, calls for patient-centered research approaches in valvular heart disease were launched by diverse professional societies and working groups [4, 5].

Patient anxiety affecting about 37% of patients with AS is often unintentionally neglected by consenting physicians [6]. Anxiety significantly impacts on patients’ decision process and may, in the extreme, lead to refusal of the indicated procedure [7]. Several other factors may pose significant limitations to the shared decision-making [3]: advanced age, comorbidities, and impairment of cognitive function [8] as well as treatment-related factors such as the procedural complexity of the TAVI intervention.

Data on the effectiveness of using alternative IC approaches are sparse. In a pilot project, we demonstrated that the use of an illustrative information brochure before coronary angiography and percutaneous coronary intervention [10] significantly improved patient-centered outcomes [11]. We hypothesized that medical illustrations may be of particular benefit to inform elderly, cognitively impaired patients about TAVI and positively impacts on understanding and periprocedural anxiety. The prevalence of AS and the number of TAVI procedures are expected to significantly increase over the next years [4]. Using medical graphics may be useful to overcome the well-known limitations of the current IC practice for a growing number of patients scheduled for complex procedures such as TAVI.

## Methods

### Study design and study population

For this prospective, randomized controlled dual center trial we consecutively screened 315 patients between February 2021 and November 2021 that were scheduled for elective TAVI at the Cardiovascular Core Unit (CVCU), Charité—Universitätsmedizin Berlin, and the Department of Cardiothoracic and Vascular surgery, Deutsches Herzzentrum Berlin (DHZB), Germany (both institutions merged on January 1st 2023 to form the Deutsches Herzzentrum der Charité, Berlin, Germany), for study participation. Patients scheduled for elective TAVI procedure who were aged > 18 years and willing to participate in the trial were included in the study. Exclusion criteria were planned interventions aside of the aortic valve, severe psychiatric disorders or serious cognitive dysfunction, insufficient ability to read or speak German, and lack or withdrawal of study consent. All patients gave verbal and written informed consent for study participation. The study was reviewed and approved by the institutional ethics committee of the Charité—University Medicine Berlin (registration number EA1/139/20). The trial is registered with the German Registry of Clinical Studies (DRKS00021661).

### Randomization and masking

Patients were randomized in a 1:1-fashion to receive either a booklet containing general information about the hospital Charité—Universitätsmedizin Berlin (Control group) or a patient comic brochure (Comic group) that illustrates central IC-related aspects by using medical graphics and simple text. Randomization was done by using tables of random numbers that were generated by stochastic computer-based allocation. All patients were informed about the procedure by the same study physician. Delivering the standard IC (official legal written information sheet and conversation with a physician) took place before randomization to avoid information bias through the physician delivering the IC.

### Intervention and procedures

**Fig. 1:**
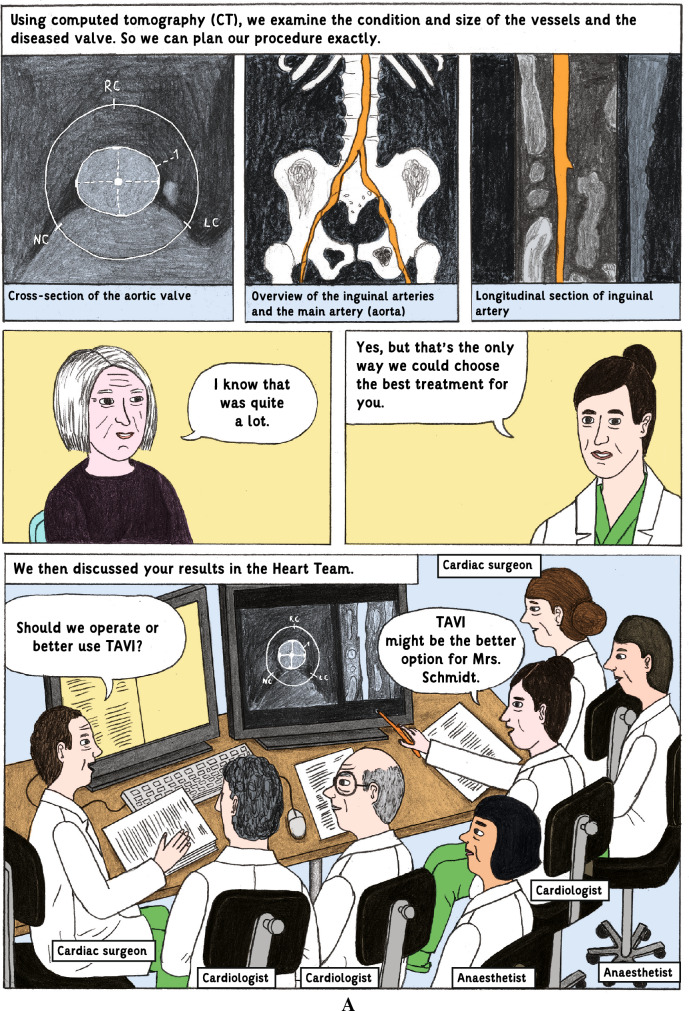

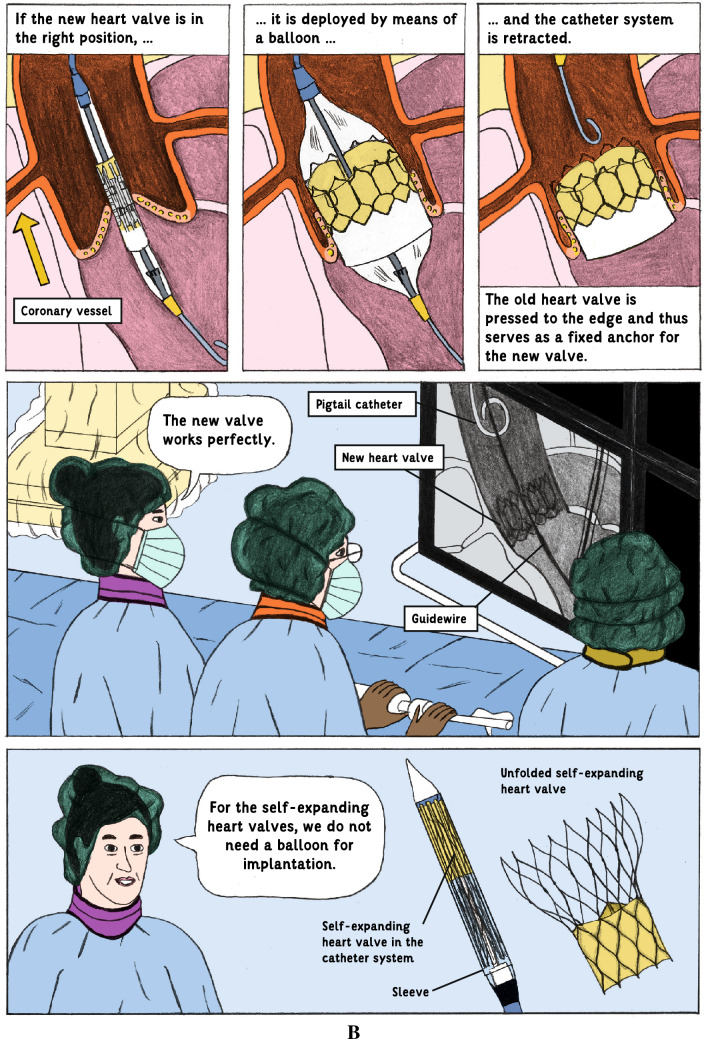
Pages 7 (Fig. 1A) and 12 (Fig. 1B) of the graphic-based patient information brochure which describes on 22 pages central informed consent-related aspects such as the single steps of the procedure including diverse access sites for valve deployment and types of prostheses, risks, benefits, treatment alternatives, and behavioral measures after the procedure by using medical graphics and simple text

The intervention consisted of an additional information brochure containing medical graphic narratives to inform about the planned procedure. The illustrative IC brochure was created on purpose for this study in collaboration with an agency for science communication (“mintwissen”) and a professional illustrator. This brochure illustrates on 22 pages central IC-related aspects such as the single steps of the procedure including diverse access sites for valve deployment and types of prostheses, risks, benefits, treatment alternatives, and behavioral measures after the procedure by using medical graphics and simple text (see example, Fig. 1; and Supplement for the whole graphic-based information brochure). In the Control group, an excerpt of a booklet containing general information about the Charité—Universitätsmedizin Berlin was used instead as additional reading material (sham intervention) as part of the IC process. After the screening visit and obtaining the patients’ IC to participate in the study, patients were handed a questionnaire assessing demographic and clinical characteristics, baseline state anxiety according to the validated Spielberger Trait Anxiety Inventory (STAI), a simplified version of the Big-Five-Inventory-10 [BFI-10] short-form to investigate patients’ individual anxiety predisposition, as well as cognitive function according to the Montreal Cognitive Assessment (MoCA). We chose the MoCA due to its broad application as a validated test with a high sensitivity to diagnose also subclinical declines of cognitive function [12].

All patients were first informed about the planned TAVI procedure by using the standard IC approach, i.e., obtaining the official legal written patient information and consent form (*Thieme Compliance, Diomed Info Kard 9*) for reading and a conversation with the consenting physician who provided detailed information about the procedure, risks and benefits, behavioral measures, and treatment alternatives. If randomized to the Comic group, patients were then handed the information brochure containing medical graphics as additional IC material. Patients in the Control group, on the other hand, were asked to read parts of a general information booklet of the hospital. Questionnaires investigating our endpoints were filled out by the patients in both groups after a time period of 60 min (paper pencil form).

### Outcomes

Primary outcomes of the study were patient comprehension of central IC-related aspects and periprocedural state anxiety across time points. To test patient comprehension, we designed a multiple choice (MC) questionnaire containing 14 simple questions about relevant aspects of the disease, procedural details, risks, treatment alternatives and behavioral measures (see Supplement for all questionnaires). Answers to these 14 items (3 answering options for each question) were scored as correct/incorrect, resulting in a sum range of 0 (minimum) to 14 (maximum score). Patient comprehension was then analyzed according to the cognitive function as assessed by the MoCA. Applying the classification of Nasreddine et al. [12] to our study sample, we defined two MoCA categories: No cognitive dysfunction (MoCA score 26–30) and impaired cognitive function (MoCA score < 26).

State anxiety was tested using the validated STAI before (T1) and after (T2) the IC procedure in order to assess the effect of our intervention on periprocedural anxiety. The STAI score consists of 20 questions with a rating scale from 1 to 4 (maximum anxiety level 80, minimum anxiety level 20). This score is widely used to assess state and trait anxiety. Mean values of working adults are described to be 35.72 ± 10.40 for males, and 35.20 ± 10.61 for women while in general medical and surgical patients, mean state anxiety values are reported to be as high as 42.68 ± 13.76 [13]. To adjust the different STAI scores at T1 and T2 for patients’ individual anxiety predisposition (trait anxiety), we used the score of a simplified version of the Big-Five-Inventory-10 (BFI-10; see patient questionnaire in Supplement for details) as a covariate.

### Statistical analysis

Case number calculations were based on the effect range seen in our pilot study [11] providing proof-of-concept data on the utility of using medical graphic narratives to assist the patient IC. While we found medium to large effects for patient comprehension and periprocedural anxiety [11], we took into account the uncertainty of the estimation and used the lower limit of the 90% confidence interval around the smallest effect for an a priori power analysis. Aiming at a minimum power of 0.8, we yielded an optimal sample size of 122 persons per patient group (Cohen’s *d* = 0.36; adjusted alpha of 0.025 for multiple testing).

Our data set was checked by an independent data manager for potential errors. Statistical analyses were performed using R (R Core Team, 2021). For patient comprehension, the effects of cognitive status, IC method and their interaction were tested as a two-factorial analysis of variance (ANOVA) within a regression modeling framework. The group differences displayed in Table 2 were based on post hoc *t* tests with Tukey adjustments to the *p* values.

For STAI, the combined effects of IC group and cognitive status across time points were investigated using a mixed design with measurement occasion as the within subject factor and trait anxiety as a covariate. Group means and differences displayed in Table 3 are based on this model. A *p* value < 0.05 was considered statistically significant.

## Results

### Study population

**Fig. 2:**
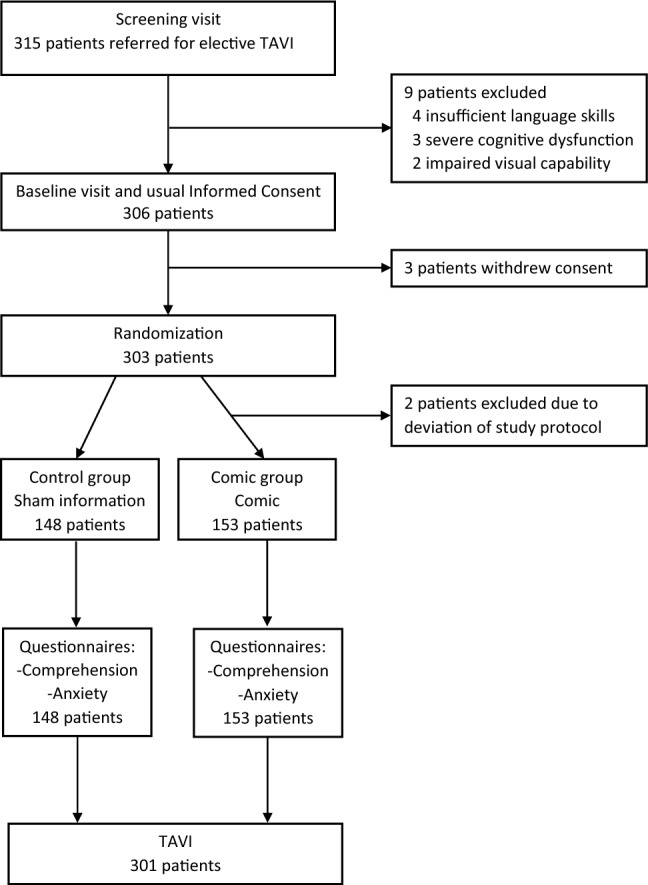
Trial profile

Between February and November 2021, we screened 315 patients scheduled for elective TAVI procedure for study participation. Four patients were excluded due to insufficient language skills, three due to severe cognitive dysfunction, two due to impaired visual capability, and three withdrew their consent to participate in the study. Two patients were excluded after randomization due to violation of study protocol (Fig. 2). Of the remaining 301 patients scheduled for elective TAVI, 153 (50.8%) were informed about the procedure by using the graphic-based information brochure in addition to standard care*.* Female study proportion of the total cohort was 46.2%. Demographic and clinical characteristics were similarly distributed in both groups (Table 1). Cognitive function was impaired (MoCA < 26) [12] in 54.8% of all patients. Mean MoCA score of the total cohort was 24.0 (SD 4.4) and was similar between groups (Table 1).

**Table 1:** Demographical and clinical characteristics of the study population

	Total (*n* = 301)	Comic group (*n* = 153)	Control group (*n* = 148)
Demographical characteristics			
Age, y	81.1 (6.8)	81.0 (6.0)	81.2 (7.4)
Female	140 (46.5%)	75 (49.0%)	65 (43.9%)
Native speaker	294 (97.7%)	149 (97.4%)	145 (98.0%)
Working in medical sector	22 (7.3%)	10 (6.5%)	12 (8.1%)
Highest education level			
No profession	19 (6.3%)	6 (3.9%)	3 (2.0%)
Professional education	204 (67.8%)	109 (71.2%)	95 (64.2%)
University or polytechnic degree	78 (25.9%)	38 (24.9%)	40 (27.0%)
Marital status			
Single	56 (18.6%)	33 (21.6%)	23 (15.5%)
In a relationship	159 (52.8%)	78 (51.0%)	8 (54.7%)
Widowed	86 (28.6%)	42 (27.5%)	44 (29.7%)
Clinical characteristics			
BMI (kg/m^2^)	27.2 (5.5)	27.3 (5.8)	27.1 (5.1)
History of syncope	39 (13.0%)	16 (10.5%)	23 (15.5%)
Dizziness	136 (45.2%)	75 (49.0%)	61 (41.2%)
Heart failure symptoms according to NYHA classification			
NYHA I	61 (20.3%)	29 (19.0%)	32 (21.6%)
NYHA II	157 (52.2%)	86 (56.2%)	71 (48.0%)
NYHA III	58 (19.3%)	28 (18.3%)	30 (20.3%)
NYHA IV	25 (8.3%)	10 (6.5%)	15 (10.1%)
Angina pectoris according to CCS classification			
CCS 1	202 (67.1%)	101 (66.0%)	101 (68.2%)
CCS 2	65 (21.6%)	35 (22.9%)	30 (20.3%)
CCS 3	17 (5.6%)	9 (5.9%)	8 (5.4%)
CCS 4	17 (5.6%)	8 (5.2%)	9 (6.1%)
Cognitive function			
Normal (MoCA score ≥ 26)	136 (45.2%)	78 (51.0%)	58 (39.2%)
Impaired (MoCA score < 26)	165 (54.8%)	75 (49.0%)	90 (60.8%)
MoCA score	24.0 (4.4)	24.8 (3.6)	23.1 (4.9)

Continuous variables are represented as mean (standard deviation), Categorial variables as numbers (%)

*BMI* body mass index, *NYHA* New York heart failure association, *CCS* Canadian cardiovascular society, *MoCA* montreal cognitive assessment

### Patient comprehension

The mean number of correct answers in the IC-related multiple choice-questionnaire was 12.8 (SD 1.2) in the Comic group and 11.3 (1.8) in the Control group [mean difference 1.5 (95% CI 1.2–1.8); *p* < 0.001; Table 2]. Mean differences of correct answers between IC groups in the presence of normal and impaired cognitive function were 1.0 (95% CI 0.6–1.5) and 1.8 (1.3–2.2) (*p* < 0.001, Table 2). In our two-factorial ANOVA, the main effects of IC method and cognitive status were significant (< 0.001 each), as was the two-way interaction between IC method and cognitive status (*p* for IC method x MoCA category interaction = 0.017; Table 2, Graphical Abstract).

**Table 2:** Patient comprehension and anxiety according to informed consent (IC) group

	Comic group (*n* = 153)	Control group (*n* = 148)	Mean difference (95% CI)	*p*
Patient comprehension, expressed as mean (SD) of correct answers				
Total score (/14)	12.8 (1.2)	11.3 (1.8)	1.5 (1.2–1.8)	< 0.001
MoCA ≥ 26	13.0 (1.0)	12.0 (1.3)	1.0 (0.6–1.5)	< 0.001
MoCA < 26	12.6 (1.2)	10.9 (1.6)	1.8 (1.3–2.2)	< 0.001
Periprocedural anxiety across measuring time points, expressed as mean STAI score (SD)				
STAI score before IC	40.9 (5.4)	40.3 (6.1)	0.6 (− 0.7 to 1.9)	0.384
STAI score after IC	35.2 (4.7)	39.5 (6.1)	− 4.3 (− 5.5 to − 3.1)	< 0.001

*MoCA* montreal cognitive assessment, *STAI* Spielberger state trait anxiety inventory, *IC* informed consent

### Periprocedural anxiety

All two-way interactions were significant, as was the three-way interaction between IC method, cognitive function, and measurement time point (*p* for IC method x measuring time point < 0.001; *p* for IC method x MoCA category x measuring time point interaction = 0.018). Table 3 and the Graphical Abstract show the differences between groups in STAI scores across measuring time points. Mean STAI score decreased by 5.7 points (95% CI 5.1–6.3; *p* < 0.001) in the Comic and by 0.8 points (0.2–1.4; *p* = 0.015) in the Control group. Notably, state anxiety declined after the IC in the Comic group, both among patients with and without cognitive impairment: Mean STAI score decrease in patients without cognitive impairment was 6.6 (95% CI 5.8–7.5; *p* < 0.001) in the Comic and 0.7 (− 0.3 to 1.7; *p* = 0.176) in the Control group. In the presence of cognitive impairment, mean STAI score decrease was 4.7 (95% CI 3.8–5.6; *p* < 0.001) and 0.9 (0.1–1.7; *p* = 0.028) in the Comic and Control group (Table 3; Graphical Abstract).

**Table 3:** Periprocedural anxiety before and after the informed consent (IC)

	Before IC	After IC	Mean difference (95% CI)	*p*
Periprocedural anxiety, expressed as mean STAI score (SD)				
Total cohort	40.6 (5.4)	37.3 (5.7)	3.3 (2.8–3.7)	< 0.001
Periprocedural anxiety in the Comic group, expressed as mean STAI score (SD)				
Total (*n* = 153)	40.9 (5.4)	35.2 (4.7)	5.7 (5.1–6.3)	< 0.001
MoCA ≥ 26 (*n* = 78)	41.6 (4.6)	34.9 (3.7)	6.6 (5.8–7.5)	< 0.001
MoCA < 26 (*n* = 75)	40.1 (5.1)	35.4 (5.2)	4.7 (3.8–5.6)	< 0.001
Periprocedural anxiety in the Control group, expressed as mean STAI score (SD)				
Total (*n* = 148)	40.3 (6.1)	39.5 (6.1)	0.8 (0.2–1.4)	0.015
MoCA ≥ 26 (*n* = 58)	39.1 (5.9)	38.4 (5.9)	0.7 (− 0.3 to 1.7)	0.176
MoCA < 26 (*n* = 90)	41.1 (5.9)	40.2 (5.9)	0.9 (0.1–1.7)	0.028

Means conditional on trait anxiety

*IC* informed consent, *STAI* Spielberger state trait anxiety inventory, *MoCA* montreal cognitive assessment

To sum up, STAI scores significantly decreased after the IC procedure in the Comic group in both MoCA categories, indicating the potential of medical graphics to mitigate periprocedural anxiety both in TAVI patients with and without cognitive impairment.

## Discussion

This study is the first prospective, randomized controlled trial investigating potential benefits of medical graphics on top of usual IC practice in elderly patients scheduled for elective TAVI and provides several important observations: (1) Medical graphic narratives considerably improved patient comprehension of central IC-related aspects and attenuated periprocedural anxiety compared to usual patient care. (2) The beneficial effect of medical graphics on patient comprehension was more pronounced in patients with cognitive dysfunction. (3) The favorable effect of medical graphics on periprocedural anxiety was similar in patients with and without cognitive impairment.

Current ESC/EACTS guidelines for the management of valvular heart disease highlight the importance of a patient-centered approach when informing about the planned procedure [3]. The majority of treating physicians, however, appear to be unfamiliar with the concept of the shared decision-making and, consequently, the principles of informing and involving patients according to their individual limitations and values are still alarmingly neglected in clinical practice [9, 14, 15]. Several individual factors may additionally impede the patient's full understanding before complex technical procedures such as TAVI: Cognitive function declines with increasing age [8], especially in patients with cardiovascular diseases [16, 17]; in addition, the majority of the elderly population has reportedly only limited knowledge about aortic stenosis with low awareness of the high prevalence and burden of valvular heart disease [18]. At the same time, in semi-structured interviews asking patients about their preferences and wishes with regards to the scheduled TAVI procedure, patients demonstrated a clear information-seeking aspiration, requesting optimal patient-physician communication, adequate information about the procedure, as well as measures to reduce anxiety [6]. However, data from randomized controlled trials that focus on the individual limitations, needs, and preferences regarding the patient IC expressed by the elderly TAVI population are so far lacking. We aimed to address this important research gap by delivering evidence derived from a prospective, randomized controlled trial that (1) substantiates the limitations of the current IC practice before TAVI, (2) suggests a significant benefit of using medical graphics to improve patient understanding, especially in patients with cognitive impairment; and (3) demonstrates a marked decrease of periprocedural anxiety by using medical graphics.

Visualization of health-related data is an effective tool to communicate complex information to broad audiences, patients and their relatives, and is also applied as a teaching format in medical education [19, 20]. Providing graphics in a sequential narrative format to communicate complex health-related information may offer particular benefits: The simultaneous cognitive processing of narrative verbal und visual information has been recognized to improve patient understanding [11], to render information to be more engaging, interesting and memorable [20, 21], and to decrease anxiety [11]. These effects may hence stimulate the affective involvement of readers. All these benefits combined may enhance, compared to exposition to spoken or written words alone, patient comprehension of health-related data and mitigate periprocedural anxiety through personal identification with persons and situations.

### Limitations

Medical graphics is one tool to visualize complex medical data. Other visualization concepts such as video-based and interactive formats have been developed for patient care, and their effects need to be compared to those of medical graphics by future randomized studies. This may enable to personalize not only treatment strategies but also the way patients are informed about planned procedures in future. Four out of 315 patients could not be included in our study due to insufficient language skills that impeded full comprehension of the oral and written study information. These patients, however, might benefit most from medical graphics. Future studies on the effectiveness of alternative information approaches are warranted to address the potential benefit of easy and understandable information material in these patients. Another limitation inherent to current patient care needs to be taken into account: while data visualization concepts have shown to effectively improve patient knowledge, their use may be limited by the caring physicians themselves who may be reluctant to changing their common clinical routine [9]. Continuous efforts of health care providers and responsible medical educators are necessary to promote the paramount importance of understandable and effective IC methods with proven benefits on patient-centered endpoints.

## Conclusion

Our data show beneficial effects of using medical graphics to inform elderly patients about the planned TAVI procedure. Medical graphic narratives significantly improved patient comprehension, with a more pronounced benefit in patients with cognitive impairment, and mitigated periprocedural anxiety. This information format may therefore be of particular interest for future individualized IC approaches in the elderly population.

## Data Availability

Data can be obtained for research purposes upon request to the corresponding author.

## Abbreviations

AS: Aortic valve stenosis
TAVR: Transcatheter aortic valve replacement
IC: Informed consent
STAI: Spielberger state trait anxiety inventory
MoCA: Montreal cognitive assessment
